# Loss of SIRT3 leads to a compensatory shift in cellular metabolism promoting cancer cell growth

**DOI:** 10.1186/2049-3002-2-S1-O18

**Published:** 2014-05-28

**Authors:** Eoin McDonnell, Olga R Ilkayeva, Robert D Stevens, Michael J Muehlbauer, James R Bain, Tomas C Becker, Matthew D Hirschey

**Affiliations:** 1 Duke Institute of Molecular Physiology, Sarah W. Stedman Nutrition and Metabolism Center, and Department of Medicine, Duke University Medical Center, Durham, NC, USA

## Background

Mechanisms involved in regulating metabolic reprogramming in cancer cells are not fully understood. Acetylation is emerging as a major regulator of mitochondrial metabolism and may contribute to metabolic derangements that occur in cancer cells. Sirtuin-3, (SIRT3), is the main mitochondrial deacetylase and it serves to maintain mitochondrial energy homeostasis by deacetylating and activating mitochondrial proteins. Loss of SIRT3 leads to altered cellular metabolism including reduced ATP production and decreased fatty acid oxidation [1]. Remarkably, reduced SIRT3 expression is associated with cancer in patients and Sirt3 knockout mice [2]. However, the mechanism of mitochondrial protein hyperacetylation and the sub-sequent increased susceptibility to tumor formation remains unknown.

## Methods

Stable cell lines expressing shScramble or shSIRT3 were made by lentiviral transduction in HepG2 and 293T cells. Metabolomic analysis of stable cell lines were done by GC/MS and MS/MS. Glutamine oxidation was measured by treating cells with 14C glutamine and capturing radiolabeled CO2.

## Results

Protein acetylation is particularly sensitive to nutrient status, such as high fat diet feeding [3]. Our data show that under the stress of a high fat diet, Sirt3 KO mice are significantly more susceptible to the development of spontaneous hepatocellular carcinoma (92% penetrance) than wild type mice (26% penetrance) after 12 months. Here we show metabolomics data that suggests that SIRT3 in HepG2 cells increased glutamine metabolism indicated by lower relative glutamine levels, and altered alpha-ketoglutarate levels. In support of this we find that SIRT3 knockdown cells have increased glutamine oxidation, a characteristic metabolic change in cancer cells. Additionally, these cells show increased expression of glucose transporter, GLUT1, increased glucose uptake, and elevated levels of citrate and other markers of increased lipid synthesis.

## Conclusions

These data illustrate an anabolic shift in cell metabolism that is required to supply biosynthetic precursors necessary for rapid cell growth. We believe that loss of SIRT3 and subsequent hyperacetylation of mitochondrial proteins leads to a rerouting of energetic substrates supporting oncogenically driven tumor growth.
